# Sexual harassment, other types of harassment and derogatory treatment in the academy

**DOI:** 10.1093/eurpub/ckac131.270

**Published:** 2022-10-25

**Authors:** F Pilgaard, A Agardh, P-O Östergren, G Priebe

**Affiliations:** 1 Division of Social Medicine and Global Health, Lund University, Malmö, Sweden; 2 Department of Social and Psychological Studies, Karlstad University, Karlstad, Sweden

## Abstract

**Background:**

Sexual harassment (SH) continues to be a significant public health problem, especially among women. SH and other types of harassment and derogatory treatment/bullying exist at many academic workplaces. The aim of this study is to explore how SH relates to other forms of harassment among staff at a large Swedish university, separated by gender.

**Methods:**

Using data from a web-based survey sent out to all staff in November 2019 (response rate 33%), a multiple logistic regression analysis was performed. Exposure to SH was defined as having experienced at least one of ten defined SH behaviours related to work. Exposure to harassment (other than sexual) was defined as having experienced violation of onés dignity associated with one of the Swedish seven legal grounds for discrimination: sex, transgender identity or expression, ethnicity, religion or other belief, disability, sexual orientation or age. Exposure to derogatory treatment was defined as having experienced undesirable negative behaviours, such as withholding information, derogatory comments or exclusion. All SH, harassment and derogatory treatment took place during the last 12 months.

**Results:**

Preliminary results show a sixfold increased risk among women subjected to SH to also experience harassment, a three times higher risk to experience derogatory treatment and a seven times higher risk to experience multiple forms of harassment (two or more forms of harassment or derogatory treatment) compared to women unexposed to SH. The elevated risk remained after adjusting for relevant background variables. The pattern was similar among men but with lower prevalence of SH, harassment and derogatory treatment.

**Conclusions:**

The results indicate that individuals subjected to SH at work have an increased risk of experiencing other types of harassment or derogatory treatment. This new information is relevant to consider in prevention of SH and harassment in academia.

**Key messages:**

• The results indicate that individuals subjected to sexual harassment at work have an increased risk of experiencing other types of harassment or derogatory treatment.

• Findings indicating a relationship between sexual harassment and other types of harassment or derogatory treatment may be valuable for counteracting the problem.

